# Irisin and Cardiometabolic Disorders in Obesity: A Systematic Review

**DOI:** 10.1155/2023/5810157

**Published:** 2023-10-19

**Authors:** Jorge da Silva Pinho-Jr, Flávio Andrade Camacho, Carollyne dos Santos Cavararo, Paula Ferreira Baião, Renata Frauches Medeiros, Sérgio Girão Barroso, Andrea Cardoso de Matos

**Affiliations:** ^1^Postgraduate Program in Nutrition Sciences, Faculty of Nutrition Emília de Jesus Ferreiro, Fluminense Federal University, Niterói, Rio de Janeiro, Brazil; ^2^Systemic Arterial Hypertension Research Center (NuPHAS/UFF), University Hospital Antonio Pedro (HUAP), Fluminense Federal University, Niterói, Rio de Janeiro, Brazil; ^3^Nutrition and Dietetics Department, Faculty of Nutrition Emília de Jesus Ferreiro, Fluminense Federal University, Niterói, Rio de Janeiro, Brazil; ^4^Postgraduate Program in Cardiovascular Sciences, Faculty of Medicine, Fluminense Federal University, Niterói, Rio de Janeiro, Brazil

## Abstract

**Background:**

Overweight and obesity are global health issues, impacting a significant portion of young adults. Obesity is a complex condition influenced by genetics and environmental factors, leading to increased susceptibility to cardiovascular diseases (CVDs), hypertension, dyslipidemia, and insulin resistance. Irisin, a protein derived from the cleavage of fibronectin type III domain-containing protein 5, may have relationship with these cardiometabolic diseases.

**Objective:**

This systematic review aims to examine the relationship between serum irisin levels and obesity, particularly in individuals predisposed to cardiovascular risk factors.

**Methods:**

A thorough literature search was conducted in multiple databases, including “Science Direct,” “Scopus,” “PubMed,” and “Lilacs,” from July 2020. Inclusion criteria encompassed subjects with metabolic disorders (with or without obesity, BMI ≥30 kg/m^2^), clinical trials, and observational studies published between 2010 and June 2020. Exclusion criteria were animal studies, meta-analyses, systematic reviews, studies evaluating only healthy subjects, and those investigating disorders beyond cardiometabolic diseases.

**Results:**

Out of 151 identified articles, 30 met the inclusion criteria. These studies, published between 2013 and 2020, assessed adults (≥21 years) and included 26 observational studies and 4 clinical trials (*n* = 7585 subjects). All studies examined irisin's role in obesity and CVDs, often including associated diseases such as type 2 diabetes and hypertension. Despite varying sample sizes, the samples within the articles were homogeneous. Observational studies exhibited a low risk of bias in at least 60% of the evaluated domains. Clinical trials demonstrated a low risk of bias in at least 50% of the domains. *Limitations*. Although the systematic review provides valuable insights, it is limited by the available literature and the varying methodologies used across studies.

**Conclusion:**

The review suggests that irisin plays a significant role as both a preventive measure and a biomarker for comorbidities linked to obesity and cardiometabolic disorders. Future research should focus on standardized irisin measurement methods and diverse populations to further elucidate its mechanisms of action.

## 1. Introduction

Overweight and obesity, according to the World Health Organization, stand as predominant global nutritional and metabolic disorders, impacting approximately 39% and 13% of young adults in multiple countries, thereby reflecting their widespread prevalence [1]. Obesity is characterized as a metabolic and endocrine ailment, stemming from the intricate interplay between genetic predisposition and environmental factors conducive to excessive weight gain [2]. This condition substantially escalates the susceptibility to cardiovascular diseases (CVDs) while adversely influencing blood pressure and overall cholesterol profiles. Moreover, it fosters insulin resistance, thereby hindering the efficient uptake of glucose. As a result, metabolic dysfunctions emerge as prominent contributors to the onset and progression of various chronic noncommunicable diseases, encompassing type 2 diabetes mellitus, dyslipidemia, and select malignancies [3, 4].

Two distinct types of adipose tissue exist within the human body, namely, the white adipose tissue and brown adipose tissue, each bearing unique metabolic functions. The white adipose tissue predominantly serves as an energy reservoir and is notorious for its role in expressing inflammatory cytokines, with potential implications for immune function. In contrast, the brown adipose tissue assumes a metabolic and thermogenic role, primarily owing to the expression of uncoupling protein 1 (UCP1) or thermogenin—a mitochondrial protein that augments thermogenesis, culminating in heightened energy expenditure. Emerging research hints at an inverse correlation between the brown adipose tissue and obesity-associated comorbidities, thereby underlining its potential in mitigating metabolic dysfunction [5–8].

Irisin, an intriguing myokine, surfaced as a novel hormone in 2012, with its origins rooted in muscle tissue. Its regulation is orchestrated by the peroxisome proliferator-activated gamma coactivator1-alpha receptor (PGC-1*α*), a transcriptional coactivator spurred into action by physical exercise in muscle tissue. PGC-1*α* catalyzes the expression of the fibronectin type III domain-containing protein 5 (FNDC5) gene, culminating in the synthesis of FNDC5 protein, which is subsequently cleaved to yield irisin, released into the bloodstream [9]. Irisin is postulated to modulate human adipocytes, facilitating the induction of brown adipocyte-like characteristics. This “browning” process endows adipocytes with heightened thermogenic potential, effectively curbing the risk of CVDs [10].

Studies conducted by Sanchis-Gomar and Perez-Quilis [11] have underscored irisin's role in ameliorating insulin resistance, thereby enhancing glucose and lipid metabolism. This implies that irisin may hold the potential to bolster metabolic regulation. Similarly, research by Chen et al. [12] has provided corroborative evidence, suggesting that elevated irisin levels are inversely associated with hepatocyte triglycerides, potentially delaying the progression of liver-related ailments. Nevertheless, the precise mechanisms governing irisin's effects on hepatocytes remain insufficiently elucidated.

It is posited that irisin plays a pivotal role in stimulating UCP1 expression, consequently promoting thermogenesis, elevating energy expenditure, aiding in weight management, refining body composition, and regulating metabolic processes. In addition, irisin exhibits the potential to deter and postpone metabolic disorders as well as CVDs [13, 14].

However, despite the extant literature highlighting the interplay between irisin and cardiometabolic disorders, a comprehensive understanding of the underlying mechanisms remains elusive. Clinical studies investigating the causal links within this relationship remain limited. Consequently, a rigorous literature review is imperative to gain deeper insights into irisin's role, especially within the context of individuals afflicted by obesity and predisposed to cardiovascular risk factors. This review aims to systematically scrutinize the association between serum irisin levels and obesity while assessing its potential implications for cardiometabolic disorders.

## 2. Methods

### 2.1. Study Object

Irisin is a molecule that functions as a myokine and acts as a hormone, primarily produced in the muscle tissue. Within muscle cells, it is responsible for the cleavage of the FNDC5 protein into irisin. Currently, there are theories regarding its production in mitochondria within the adipose tissue [9].

In this study, we assessed whether there were any changes in serum irisin levels.

### 2.2. Search and Eligibility Criteria

We employed the PICOS strategy as follows: P for population, I for intervention, C for comparison, O for outcome, and S for study design, based on the PRISMA statement [15]. Keywords were defined by reviewers as “obesity,” “hypertension,” “diabetes,” “cardiovascular diseases,” and “irisin” combined with “and” connector.

“The research was conducted in September 2023 using the databases “Science Direct,” “Scopus,” “PubMed,” and “Lilacs.” The search strategies employed were “Irisin and Cardiovascular Diseases and Obesity,” “Irisin and Obesity and Hypertension,” and “Irisin and Obesity and Diabetes” for all databases. Inclusion criteria were limited to subjects presenting metabolic disorders with or without obesity (BMI ≥30 kg/m^2^), clinical trials, and observational studies, published from the year 2010 to June 2020 and without language limitations.

All animal studies, meta-analysis, systematic reviews, letters to the editor, studies evaluating only healthy subjects, and studies evaluating other disorders as primary outcomes beyond cardiometabolic diseases (chronic kidney disease, neuropathies, inflammatory diseases, autoimmune diseases, liver disease, thyroid-related diseases, cancer, pulmonary diseases, gastrointestinal disease, or severe system disease) were excluded.

The current systematic review was conducted as recommended by the state-of-the-art method Preferred Reporting Items for Systematic Reviews and Meta-Analyses (PRISMA, Supplementary Materials, PRISMA checklist) [15], and the protocol of this study was not previously registered.

## 3. Outcomes

Alterations in irisin levels in subjects with cardiometabolic disorders with or without obesity were the primary outcomes. As secondary outcomes, alterations in cardiometabolic and glycemic homeostasis markers (serum insulin, homeostatic model assessment, hemoglobin A1c, total cholesterol and fractions, and blood pressure) and anthropometric measures (weight, BMI, and waist circumference) were considered.

### 3.1. Data Extraction

Data extraction was conducted in pairs using a spreadsheet, following the protocol described by Pereira and Galvão [16], which is aligned with the PRISMA statement [15, 16].

Duplicate studies were excluded. The remaining studies were assessed based on their titles and abstracts to determine eligibility. Subsequently, two independent reviewers evaluated the selected studies against the eligibility criteria. Any disagreements were resolved through discussion between the reviewers. After postanalysis, studies that did not meet the specified criteria were excluded.

### 3.2. Risk of Bias

The risk of bias of each included study was independently certified by two reviewers (J.S.P.Jr and F.A.C.). Any disagreement was resolved by discussion between the reviewers. The clinical trial studies were analyzed according to the risk of bias based on the Cochrane Collaboration tool (RoB2, risk of bias). This tool is based on the following seven domains: bias arising from randomization process, allocation sequence concealment, blinding of participants and personnel, blinding of outcome assessment, attrition bias, reporting bias, and other biases [17].

Observational studies were analyzed based on the Cochrane Collaboration ACROBAT-NRSI tool. This tool is based on the following five main aspects: participant selection bias into the study, bias in measurement of intervention, bias due to confounding, bias in outcome measurement, and bias in selection of the reported result [18].

In both tools, the domains were evaluated individually and were classified as low risk, uncertain risk, and high risk of bias.

## 4. Results

**Figure 1:**
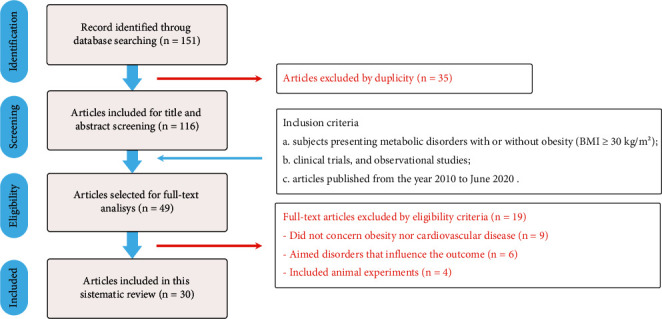
PRISMA flowchart of literature search and studies selection.

In this study, we identified 151 articles through our search. Out of these, 35 were duplicate studies and 67 were excluded based on their titles and abstracts. We thoroughly reviewed 49 studies, applying the eligibility criteria, and ultimately included 30 articles in this systematic review, as illustrated in Figure 1.

### 4.1. Studies Characteristics

Studies included in this review were published from 2013 to 2020 and evaluated adults (≥21 years). Among those, 26 were observational studies and 4 were clinical trials (*n* = 7585 subjects). All selected studies evaluated irisin concentration or functional relationship in obesity and CVD. Notwithstanding, studies evaluated subjects presenting associated diseases, such as type 2 diabetes mellitus, hypertension, and coronary-related disorders. Furthermore, the studies had different sample sizes. However, the samples presented in the articles were homogeneous. For further information on the selected articles, access Supplementary Materials Table S1.

### 4.2. Risk of Bias

Observational studies were analyzed for the risk of bias by the Cochrane Collaboration ACROBAT-NRSI tool. At least 60% of those studies were considered low risk of bias in all the domains evaluated.  Table 1 indicates that in bias in measurement of the outcome domain and in bias in selection of the reported results, over 75% of the studies were classified as low risk.

Clinical trials were evaluated by the Cochrane Collaboration risk of bias (RoB2) tool, and 50% of the studies were classified as low risk of bias in all domains.  Table 2 shows that in bias arising from the randomization process, attrition bias, and reporting bias, at least 75% of the studies were classified as low risk.

## 5. Discussion

Irisin, initially described as a myokine and hormone produced in the muscle tissue, has also been suggested to have an association with food intake in the literature. Ko et al. [38] reported that the intake of enol-rich foods, such as the Dietary Approaches to Stop Hypertension (DASH) diet, may potentially affect irisin expression. In their study, a positive correlation was observed between irisin levels and adherence to a healthy diet, as indicated by DASH scores and a prudent diet pattern. In addition, higher consumption of fruits and lower intake of meat were associated with elevated circulating irisin levels. While the study established a positive connection between irisin and dietary quality, the precise mechanisms underlying this relationship remain unclear.

There are some controversial studies suggesting that nutraceutical supplementation may not affect irisin. For example, Huerta et al. [46] demonstrated that overweight/obese subjects, who were supplemented with 300 mg/day of alpha-lipoic acid and/or 1300 mg/day of omega-3, did not experience changes in irisin levels. Interestingly, their in vitro study suggested that alpha-lipoic acid, but not omega-3, stimulated the expression of FNDC5, consequently increasing the production of irisin. In another study conducted by Agh et al. (2017) [44], subjects with coronary arterial disease were supplemented with omega-3 (720 mg eicosapentaenoic acid and 480 mg docosahexaenoic acid) for eight weeks. The study revealed an increase in irisin levels from 2.08 to 2.66 *μ*g/mL among the supplemented group, while irisin levels decreased from 2.52 to 1.85 *μ*g/mL in the control group. However, it is worth noting that the omega-3 intake in this study did not show a significant increase in the omega-3 group after intervention, and there was no statistically significant difference between the omega-3 and control groups. Therefore, without confirmation that omega-3 intake increased following supplementation, it cannot be concluded that any changes in variables are solely due to the supplementation.

The muscle tissue is primarily responsible for the expression of PGC-1*α* and the FNDC5 protein, which is cleaved into irisin. Therefore, physical activity is related to serum irisin levels, although some controversies exist regarding activity frequency and intensity. However, a study conducted by Huh et al. (2014) [48] observed an immediate increase in serum irisin levels following physical activity. This finding suggests a potential relationship between serum irisin levels and an increase in glucose and fatty acid uptake in muscle tissues, contributing to their regulation. Besides homeostasis, irisin relates to body composition through energetic expenditure. Studies have shown an inverse correlation between irisin levels and the BMI, which could be explained by differences in the body composition of the subjects evaluated. Serum irisin levels tend to show a positive correlation with the lean body mass [12, 41, 42].

Studies evaluating serum irisin levels in individuals with obesity have mostly shown a relationship between high irisin levels and a decreased risk of associated comorbidities, such as type 2 diabetes mellitus and other cardiometabolic disorders. Authors suggested that individuals with obesity and high irisin levels also had a better prognosis, lipid metabolism regulation (considering the inhibition of adenosine triphosphate synthesis and consequently heat production), as well as a lower probability of complications related to insulin resistance, reducing serum insulin levels [23, 24, 34, 37, 45]. Furthermore, high serum irisin levels negatively relate to visceral adiposity markers and could be associated with other peptide hormones, for example, follistatin [20]. A direct association among irisin, adipose tissue, and follistatin is suggested as a metabolic regulation mechanism [20, 33].

However, some studies have reported controversies regarding irisin levels in individuals with obesity [29, 30, 35]. Saaldeldin et al. [29] observed that irisin levels remained unaltered in obese patients, suggesting that it may not be a biomarker for obesity-related comorbidities. In cases of elevated irisin levels, the authors questioned whether irisin was causing the comorbidity or whether this high level could be the body's natural physiological response. Moreover, irisin levels were inversely related to age and duration of exposure to the disease. The authors developed a theory based on a probable regulatory mechanism, suggesting that tissues oversynthesize irisin in an attempt to regulate metabolism (homeostasis), causing “irisin resistance.” In those ≥60 years or with high duration of exposure to comorbidity, irisin levels were lower, which could be explained by a probable inhibition of peptide secretion caused by hyperglycemia, lower lean mass, or metabolic dysregulation [30, 35].

The role of irisin in metabolism was observed by Panagiotiou et al. [47], who linked high irisin levels to the regulation of subparticles lipoproteins and lipid metabolism. However, other studies have associated high irisin levels with the improved clinical status. Prevention of insulin resistance provided by irisin relates to lower glycated hemoglobin; thus, its reduction could also be interpreted as a biomarker of metabolic disorders [24, 31, 47]. Emanuele et al. [26] corroborated these results in a study on longevity and healthy aging. When they compared centenary subjects with young subjects with obesity, they suggested irisin could be a potential marker of the risk of CVD.

Considering the inverse correlation between irisin levels and cardiometabolic disorders, some authors have suggested irisin as a potential biomarker for these disorders. It is believed that irisin's interaction with other hormones may enhance endothelial function, possibly through tissue preservation or synergistic actions on other regulatory mechanisms related to endothelial nitric oxide synthase [32, 40].

Studies have shown an association between high irisin levels and the presence or exacerbation of cardiometabolic disorders. These disorders include cardiac cachexia, cardiac events following heart failure, or adverse cardiac events in ST-elevation myocardial infarction patients. Concerns have arisen regarding the relationship between higher irisin levels and decreased adenosine triphosphate, as adenosine triphosphate inhibition of heat production could potentially delay recovery from these disorders. However, the hyperadrenergic state induced by these disorders leads to the inhibition of glucose uptake by the muscle tissue, affecting the pancreas and exacerbating insulin resistance. This increase in insulin resistance may, in turn, lead to higher irisin levels as a compensatory mechanism, serving as a metabolic response of the adipose tissue [19, 28, 43].

Despite these findings, the majority of studies investigating the relationship between cardiometabolic disorders and irisin have suggested its potential role in preventing or delaying the progression of these comorbidities. The anti-inflammatory and hypolipidemic effects of irisin have been studied and have shown a strong correlation between high irisin levels and reduced cardiometabolic complications. This correlation even extends to the regulation of blood pressure in individuals with cardiovascular disease [25, 39].

Concerning cardiovascular diseases, low serum irisin levels could be considered biomarkers of disease progression, particularly in cases of obesity, suggesting its potential role in regulating the endothelial function. This theory was supported by Tu et al. [36], who proposed that irisin concentrations could serve as prognostic markers for infarction risk [27]. In individuals with obesity and cardiometabolic disorders, low irisin levels might be considered proarrhythmia markers, as obesity disrupts the balance in the adipose tissue and the endocrine function of the muscle tissue. Individuals with obesity and cardiometabolic complications have values up to 1.6 times higher than those with uncomplicated obesity, often referred to as “healthy obesity” [21, 22].

In their review, Singh and Rai [49] explored various factors contributing to insulin resistance and their implications for obesity and related conditions. Their comprehensive analysis encompasses topics such as endoplasmic stress and the unfolded protein response, oxidative stress, the impact of pharmaceutical agents, polycystic ovary syndrome (PCOS), leptin resistance, catecholamine resistance, mitochondrial dysfunction, hypoxia, and adipose tissue remodeling. These factors are underscored for their significance in both insulin resistance and the management of obesity. Consequently, there exists a multitude of potential confounding variables associated with obesity and a range of metabolic alterations that can influence irisin levels. Therefore, the meticulous control of these confounding factors proves indispensable in clinical trials aimed at authentically elucidating the association between obesity and irisin expression. As expected, disparities were found in the analyzed studies.

Moreover, another review provides valuable insights into the relationship between microbiota and obesity, shedding light on the various factors that impact this microbiota. The rejuvenation of microbiota can commence through dietary adjustments, but achieving a fully restored microbiota pattern necessitates not only physical changes but also mental adaptations and dietary constraints. This underscores the remarkable adaptability of the human microbiome [50].

Weight loss, which is influenced by biomarkers and the modulation of physiological responses, is a gradual process intricately linked to the restoration of microbiota. The equilibrium of the gut-brain axis emerges as a pivotal factor in achieving homeostasis by regulating neurotransmitters. Notably, dietary bioactive compounds, especially polyphenols, show potential in this context. However, their clinical significance awaits confirmation through further in vitro and in vivo studies [50].

It is important to note that irisin is a relatively new area of study, and due to the limited available information, the selected studies, despite sharing general similarities, primarily investigated specific populations in various regions with diverse cultures and behaviors. These regional and cultural differences directly influenced their results, considering the influence of dietary patterns in these distinct scenarios.

## 6. Conclusion

Based on the studies we have analyzed, it can be concluded that irisin is a significant hormone with roles in both preventing and serving as a biomarker for comorbidities associated with obesity and cardiometabolic disorders. It is recommended that standardized methods for measuring irisin levels should be validated. Furthermore, future research should encompass diverse populations to gain a clearer understanding of the mechanisms underlying irisin's actions.

## Figures and Tables

**Table 1:** Analysis of the risk of bias of observational studies with the ACROBAT-NRSI tool.

Authors (years)	Participants selection bias	Bias in measurement of interventions	Bias due to confounding	Bias in outcome measurement	Bias in selection of reported results
Kalkan et al. (2018) [19]	Low risk	Low risk	Low risk	Low risk	Low risk
Vamvini et al. (2013) [20]	Uncertain risk	Low risk	Uncertain risk	Low risk	Low risk
Anaszewicz et al. (2019) [21]	Uncertain risk	Low risk	High risk	Uncertain risk	Low risk
El-Lebedy et al. (2018) [22]	Low risk	Low risk	Low risk	Low risk	Low risk
Bonfante et al. (2017) [23]	Uncertain risk	Uncertain risk	Uncertain risk	High risk	Uncertain risk
Belviranli et al. (2016) [24]	Uncertain risk	Low risk	Low risk	Low risk	Low risk
Abd El-Mottaleb et al. (2019) [25]	Low risk	High risk	Uncertain risk	Low risk	Low risk
Emanuele et al. (2014) [26]	Low risk	Low risk	Low risk	Low risk	Low risk
Deng (2016) [27]	Uncertain risk	Low risk	Uncertain risk	High risk	Low risk
Shen et al. (2017) [28]	Low risk	Low risk	Low risk	Low risk	Low risk
Saadeldin et al. (2018) [29]	Low risk	Low risk	Low risk	Uncertain risk	Low risk
Khorasani et al. (2019) [30]	Low risk	Low risk	Low risk	Low risk	Low risk
Tang et al. (2019) [31]	Low risk	Uncertain risk	Uncertain risk	Low risk	Low risk
Hou et al. (2015) [32]	Uncertain risk	Low risk	Uncertain risk	Low risk	Low risk
Campolo et al. (2020) [33]	Low risk	Uncertain risk	Low risk	Low risk	Low risk
Sahin-Efe et al. (2018) [34]	Low risk	Low risk	Uncertain risk	Low risk	Low risk
Sesti et al. (2014) [35]	Low risk	Low risk	Uncertain risk	Low risk	Low risk
Tu et al. 2018 [36]	Low risk	Low risk	Low risk	Uncertain risk	Low risk
Shi et al. (2016) [37]	Uncertain risk	Low risk	Low risk	Uncertain risk	Low risk
Ko et al. (2016) [38]	Low risk	Uncertain risk	Low risk	Low risk	Low risk
Aronis et al. (2015) [39]	Low risk	Low risk	Low risk	Low risk	Low risk
Sanchis-Gomar and Perez-Quilis (2014) [11]	Low risk	Low risk	Low risk	Low risk	Low risk
Silvestrini et al. (2019) [40]	Low risk	Low risk	Low risk	Low risk	Low risk
Stengel et al. (2013) [41]	Uncertain risk	Low risk	Low risk	Low risk	Low risk
Shoukry et al. (2016) [42]	Low risk	Low risk	Low risk	Low risk	Low risk
Hsieh et al. (2018) [43]	Low risk	Low risk	Low risk	Low risk	Low risk

Source: own authorship.

**Table 2:** Analysis of the risk of bias of clinical trials studies with the Cochrane Collaboration risk of bias (RoB2) tool.

Authors (years)	Bias arising from randomization process	Allocation sequence concealment	Blinding of participants and personnel	Blinding of outcomes assessment	Attrition bias	Reporting bias	Other biases
Agh et al. (2017) [44]	Low risk	Low risk	Low risk	Uncertain risk	Low risk	Low risk	Uncertain risk
Liu et al. (2016) [45]	High risk	Uncertain risk	Uncertain risk	Low risk	Low risk	Uncertain risk	Low risk
Huerta et al. (2015) [46]	Low risk	Low risk	Low risk	Low risk	Low risk	Low risk	Uncertain risk
Panagiotou et al. (2014) [47]	Low risk	Uncertain risk	Uncertain risk	Low risk	High risk	Low risk	Low risk

Source: own authorship.

## Data Availability

All data used to support the findings of this study are included within the article.

## Supplementary Materials

Supplementary Materials PICO spreadsheet of the studies analyzed in this systematic review and PRISMA checklist for systematic reviews.
